# NOD2 Expression in *Streptococcus pneumoniae* Meningitis and Its Influence on the Blood-Brain Barrier

**DOI:** 10.1155/2018/7292084

**Published:** 2018-08-13

**Authors:** Ying Wang, Xinjie Liu, Qi Liu

**Affiliations:** ^1^Department of Pediatrics, Qilu Hospital, Shandong University, 107# Wen Hua Xi Road, Jinan, Shandong 250012, China; ^2^The People's Hospital in Zoucheng, 59# Qian Quan Road, Zoucheng, Shandong 273500, China

## Abstract

*Streptococcus pneumoniae* meningitis is one of the most common disorders seen in clinical practice. It is believed that the brain tissue immune injury is caused by the expression of pattern-recognition receptors (PRR) which can further induce the release of other cytokines and inflammatory cascades. The aim of this study is to investigate the expression of nucleotide-binding oligomerization domain 2 (NOD2) and inflammatory factors in rat brain tissues infected with *Streptococcus pneumoniae* and its influence on the blood-brain barrier (BBB) permeability. Rats were given an intracranial injection of *Streptococcus pneumoniae* to construct the *Streptococcus pneumoniae* meningitis rat models. The expression change curves of NOD2 and inflammatory factors at different time points (0 h, 12 h, 24 h, 48 h, and 7 d) after *Streptococcus pneumoniae* were evaluated by enzyme-linked immunosorbent assay (ELISA). Western blotting analysis and quantitative real-time polymerase chain reaction (qRT-PCR) were engaged to examine the expression of NOD2. Furthermore, the changing processes of pathological characteristics, nervous system score, cerebral oedema, and BBB permeability were observed. Our results showed that NOD2 expression began to increase in the 12 h after *Streptococcus pneumoniae* infection group, while the remaining inflammatory factors were not obviously increased. Meanwhile, the levels of NOD2, as well as inflammatory factors IL-1*β*, TNF-*α*, and IL-6 were markedly elevated in 24 h and 48 h infection groups, which were consistent with the increases in BBB permeability and BWC, and the positive expression of NOD2 in the infected rat brain tissues was observed using immunohistochemistry (IHC). This study suggests that NOD2 might be related to the activation of inflammation pathways and the damage to the blood-brain barrier. NOD2 and inflammatory factors have played vital roles in the pathogenesis of *Streptococcus pneumoniae* meningitis.

## 1. Introduction

*Streptococcus pneumoniae* is the common central nervous system infectious pathogen in clinical practice [1, 2]. The overall mortality and prognosis for survivors remain unsatisfactory regardless of the application of effective antibiotics and the promotion of effective vaccination [3, 4]. It is currently believed that pattern-recognition receptors (PRR) are expressed and maybe produce inflammatory factors initiating the brain tissue immune injury [5]. Inflammatory factors can lead to secondary immune responses, brain oedema, and BBB dysfunction. Among all the pathomechanisms of cerebral injury, the effects of the NLRs protein family have attracted high attention in recent years [6]. So far 20 members of the NOD family have been reported, such as apoptotic protease-activating factor-1 (APAF-1), nucleotide-binding oligomerization domain 1 (NOD1), NOD2, and disease-resistance proteins (R-protein), most representative of which is NOD1 and NOD2 [7–9]. The difference between NOD1 and NOD2 is that the caspase-associated recruitment domain (CARD) of the amino-terminal domain [10, 11]. NOD1 and NOD2 proteins can identify peptidoglycan, but their recognition dose not overlap: the NOD2 protein can recognise GlcNAc-MurNAc tripeptide muropeptide (GM-Tri_lys)_ only in Gram-positive bacteria, except for identifying GlcNAc-MurNAc dipeptide (GM-di) [6]. In this section, we focused on NOD2 because *Streptococcus pneumoniae* belongs to Gram-positive bacteria.

NLRs can induce the activation of the NF-*κ*B or MAPK pathway to induce inflammatory reaction [13, 14]. Typically, NOD2 protein is involved in both of these two canonical pathways [15]. Research in vitro suggests that NOD2 protein can induce microglial cell hyperplasia and astrocyte demyelination and elevate the inflammatory cytokine levels such as the activating tumor necrosis factor (TNF) [16]. Other NLRs family members also participate in the proinflammatory effect of NOD2 in signal transduction. For instance, the intracellular pattern recognition receptor can activate the caspase ligand and apoptosis-associated speck-like protein, which will result in caspase-1 activation stimulation and IL-1*β* secretion [17]. These immune cells release proinflammatory cytokines such as tumor necrosis factor-*α* (TNF-*α*) and interleukin-1*β* (IL-1*β*), which readily cross the blood-brain barrier into the brain parenchyma and promote the immune inflammatory damage of brain tissue [16]. IL-6 is one kind of the interleukin, which is a cytokine that has been verified to be highly expressed in numerous infection models [18]. Therefore, TNF-*α*, IL-1*β*, and IL-6 were selected as the related inflammatory factors in this experiment, to further explore the expression of NOD2 as well as the related inflammatory factors in *Streptococcus pneumoniae* meningitis, as well as their influence on the BBB.

A *Streptococcus pneumoniae*-induced meningitis model was established in this study. The changing processes of pathological characteristics, the nervous system score, cerebral oedema, and BBB permeability were observed. In the meanwhile, expression change curves of NOD2 and inflammatory factors were recorded at different time points after *Streptococcus pneumoniae*. This research result provides a theoretical basis for further exploring the mechanism of NOD2 in *Streptococcus pneumoniae* meningitis.

## 2. Materials and Methods

### 2.1. Preparation of *Streptococcus pneumoniae* Suspension

*Streptococcus pneumoniae* serum type III standard bacteria were provided by the National Institute for the Control of Pharmaceutical and Biological Products (Beijing). The bacteria were inoculated on the sheep blood agarose medium under 37°C and 5% CO_2_ environments overnight. Subsequently, single colonies were selected and inoculated on the VITAL AER broth overnight. The culture solution was centrifuged and precipitated, and *Streptococcus pneumoniae* suspension at the concentration of 1.5 × 10^8^ cfu·ML^−1^ was diluted with sterile normal saline for intracisternal injection [19, 20].

### 2.2. Construction of *Streptococcus pneumoniae* Meningitis Rat Models and Organization of the Experimental Groups

Animal models were constructed according to the Papadopoulos MC method [21]. Rats were given the intraperitoneal injection of pentobarbital sodium (150 mg/kg) for anaesthesia. Briefly, rats were fixed and given posterior cistern injection, 10 *μ*L cerebrospinal fluids (CSF) was removed, and 10 *μ*L (1.5 × 10^8/ml CFU) *Streptococcus pneumoniae* was injected. Neurological impairment was assessed after intracisternal inoculation, and rats with puncture-induced neurological impairment should be excluded. This experiment was carried out in strict accordance with the China Animal Protection Act. Moreover, this research protocol was approved by the Animal Committee of Shandong University. The animals were divided into four groups of 6 h group (*n*=30), 24 h group (*n*=30) 48 h group (*n*=30), 7 d group (*n*=30), and control group (*n*=20).

### 2.3. Tissue Processing

20 rats were selected by random sampling at different stages after infection. The rats were killed at predetermined time points (at 12 h, 24 h, 48 h, and 7 d after infection) by an overdose of intraperitoneal injection of pentobarbital sodium (150 mg/kg) for anaesthesia. In brief, selected rats were given aortic perfusion of 4% ice paraformaldehyde and normal saline for fixation before they were decapitated to take the two hemispheres. The right hemispheres were fixed in 4% paraformaldehyde for 24–48 h of fixation until further processing for histopathological assessment of brain injury. Brain tissues from the left hemisphere were dissected in ice-cold phosphate buffered saline (PBS), immediately transferred into liquid nitrogen and stored at −80°C.

### 2.4. The Levels of NOD2, as well as Inflammatory Factors IL-1*β*, TNF-*α*, and IL-6 Detected by ELISA

The infected Wistar rats were randomly divided into 12 h group, 24 h group, 48 h group, and 7 d group according to the sacrifice time. Frozen brain tissues included were ground into powder under the action of liquid nitrogen, then brain tissue proteins were extracted, and the protein concentration was detected using the BCA protein concentration detection kit. The contents of IL-1*β*, TNF-*α*, IL-6, and NOD2 were detected by a commercial ELISA kit according to the directions provided by the manufacturer (Santa Cruz Biotechnology). An automatic enzyme-linked immunity analyzer was used to detect absorbance at 450 nm (SM-3, China).

### 2.5. qRT-PCR Analyses

The mRNA levels of NOD2 were determined by quantitative RT-PCR. Total RNA was extracted from the brain tissue based on the TRIzol kit instructions (Invitrogen Life Technologies, 15596-026), and then reverse-transcribed into cDNA using the RevertAid First Strand cDNA Synthesis Kit (Thermo, #K1622). The cDNA was used as the template in real-time PCR reactions to analyze the expression of NOD2. The primers in Table 1 were used in these protocols. The thermal cycling profile consisted of preincubation at 95°C for 10  min, followed by 45 cycles of 95°C for 10  s, and extension at 60°C for 60 s. The relative expression level of mRNA of NOD2 was calculated by the ΔΔCT method of GAPDH as a control.

### 2.6. Western Blot Analyses of NOD2 Level in the Brain Tissue

Brain tissues of rats were powdered in liquid nitrogen and then the cracking liquid was added (configuration by Cracking and PMSF, 100 : 1). Homogenates of brain tissues were centrifuged for 15 min (6,000 ×g, 4°C). The supernatant fluid was moved into EP tube and configured with protein buffer according to 4 : 1 ratio. The protein was heated for 10 minutes at 99°C to make protein denaturation and was detected using the BCA protein concentration detection kit (Pierce, Rockford, LA, USA). The protein was separated by sodium dodecyl sulfate/polyacrylamide gel (SDS-PAGE Bio-Rad, Hercules, CA, USA) and transferred to nitrocellulose membranes. The blotting membrane was blocked and incubated with NOD2 antibody at 1 : 1000 at 4°C overnight and incubated with secondary antibody for 1 h at 37°C. The corresponding internal reference GAPDH was used as an internal control. Protein bands were detected by densitometry analysis with Image J software.

### 2.7. Brain Tissue Section HE Staining and Immunohistochemistry (IHC)

The rats were narcotized at different time after being infected with *Streptococcus pneumoniae*. Rat skulls were fixed in the orbits using the hooked forceps, and brain tissues were collected after cutting the junctions between the skulls and cervical vertebrae using the tissue scissors. Brain tissues were then immersed and fixed in 4% paraformaldehyde for 24 h, followed by gradient ethanol dehydration, xylene transparentizing, and paraffin embedding. The immunostaining steps were shown as follows. The paraffin sections were placed into 60°C oven to roast for 1 hour, followed by xylene transparentizing, gradient ethanol dewaxing to hydration, and thermal antigen retrieval. Afterwards, NOD2 antibody at 1 : 100 (Santa Cruz Biotechnology) for 1 hour and secondary antibody (Shenda Biotechnology, Guangzhou, China) for 30 min were added dropwise after incubation, followed by sufficient washing with PBS, counterstaining, washing under cold running water, dehydration, transparentizing, and mounting. Then, the samples were observed under the microscope.

### 2.8. Nervous System Score

The neurological behaviour 5-point rating system was used to evaluate before the demise of different groups' animals, and the following evaluation indexes were used [22]. 5 points: the rats could move normally when they were caught by the back; 4 points: the rats had reduced autokinetic movement and could turn over within 5 s; 3 points: the turn over time was over 5 min; 2 points: the rats could not turn over; 1 point: the rats could not move; 0 points: the rats died.

### 2.9. Determination of BBB Permeability in Brain Tissue

Five groups (*n*=5 each group) were narcotized by an overdose of intraperitoneal injection of pentobarbital sodium (150 mg/kg), respectively. In brief, rats were given the caudal venous injection of 5% Evans blue (EB) (Sigma Aldrich, Germany) at the dose of 1 ml/kg. Left ventricular perfusion of normal saline was performed through thoracotomy 2 h after the conjunctiva, and 5 extremities in rats became blue, until colorless liquid flowed out from the right atria. The heads were cut off, the cerebral hemispheres were weighed, homogenised with 50% trichloroacetic acid, and centrifuged at 15,000  rpm for 20  min, and the optical density (OD) value was determined using the spectrophotometer (at the wavelength of *λ* = 620 nm). Evans blue content (ng/mg) in brain tissue was calculated according to the Evans blue standard curve.

### 2.10. Assessment of Brain Oedema

The head of Wistar rats in 12 h, 24 h, 48 h, and 7 d groups was cut off after deep anaesthesia, the cerebral hemispheres (*n*=5 each group) were taken, and the wet weight was weighed. Afterwards, the cerebral hemispheres were roasted in the 80°C oven until constant weight was achieved, the dry weight was weighed, and the differences between the wet weight and dry weight were the water contents. BWC (%) was calculated according to the formula = (wet weight − dry weight)/wet weight × 100%. Typically, the integrity of the weighed samples should be guaranteed.

### 2.11. Statistical Method

Data were expressed as mean ± standard deviation, and one-way analysis of variance (ANOVA) was performed to compare differences using the SPSS17.0 software. A difference of *p* < 0.05 was deemed as statistically significant.

## 3. Results

### 3.1. Characteristic of NOD2 and Its Cytokine Secretion after *Streptococcus pneumoniae* Infection

**Figure 1:**
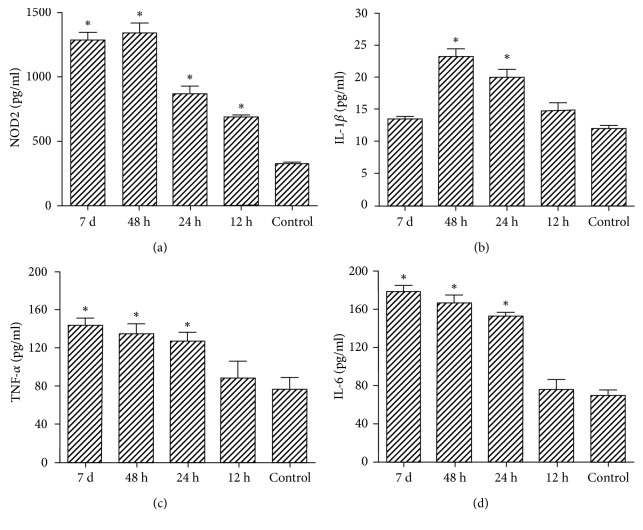
NOD2 and inflammatory factor expression levels in brain tissue at different time points (0, 12, 24, and 48 h and 7 d). Evaluated factors are NOD2, IL-1*β*, TNF-*α*, and IL-6. Data are mean ± SD from five independent experiments. The symbol (^*∗*^) indicates a significant difference versus control group considering a *p* value < 0.05.

The expressions of NOD2, IL-1*β*, TNF-*α*, and IL-6 in rat brain tissues were measured by ELISA, and their characteristics of changes were compared among the five groups. Our data suggested that NOD2 activity began to increase in brain tissue 12 h after infection, while those of IL-1*β*, TNF-*α*, and IL-6 showed no obvious changes at 12 h. Meanwhile, the expression of NOD2, as well as inflammatory factors IL-1*β*, TNF-*α*, and IL-6 was markedly up-regulated in 24 h and 48 h after infection groups. After seven days, all cytokines measured, except IL-1*β*, were at the high level (Figure 1).

### 3.2. Expression and Distribution of NOD2

The transcript levels of NOD2 were evaluated through quantitative RT-PCR at hours 12, 24, and 48 and 7 days. Compared with the normal control group, there were significant differences in the rest of the infected groups (^*∗*^*p* < 0.05). In comparison with the 48 h group, there were significant differences in control and 12 h groups. At the same time, the relative NOD2 mRNA level has no statistically significant difference in 24 h, 48 h, and 7 d groups.

**Figure 2:**
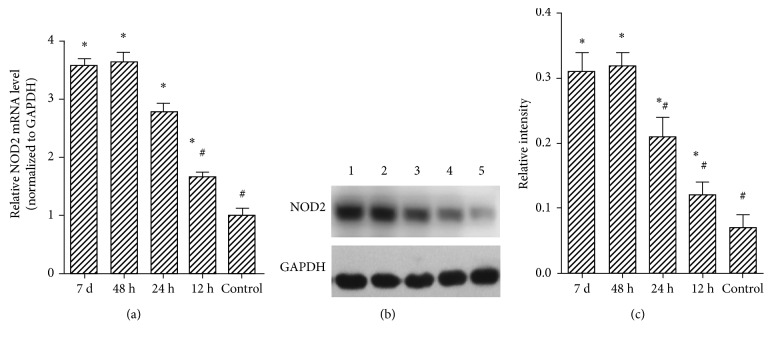
The NOD2 level analyzed by quantitative real-time polymerase chain reaction (qRT-PCR) and Western blot. (a) NOD2 level assessment based on qRT-PCR. (b) The expression level of NOD2 in different groups measured via Western blot analysis. (c) Quantitative analysis of the relative intensity of NOD2 protein. ^*∗*^ Significant difference versus the control group considering a *p* value < 0.05. ^#^ Significant difference versus the 48 h group considering a *p* value < 0.05. h: hour; d: day.

The NOD2 level in the brain tissue was measured by immunoblot (WB). One-way ANOVA revealed differences among the five groups for the NOD2 level (*p* < 0.05). The results showed that the level of NOD2 in the rat's brain homogenates began to increase at 12 h, the peak value appeared at 48 h, and NOD2 maintained at a high level throughout the experiment. Compared with the normal control group , the difference has statistical significance in the rest of the time groups. (^*∗*^*p* < 0.05). Control, 12 h, and 24 h groups of rat NOD2 levels compared with 48 h group were significantly decreased, and there was statistical significance (^#^*p* < 0.05); the 7 d group and 48 h group had no significant difference (Figure 2).

### 3.3. Brain Tissue HE Staining and Immunohistochemical Staining Sections of Wistar Rats

**Figure 3:**
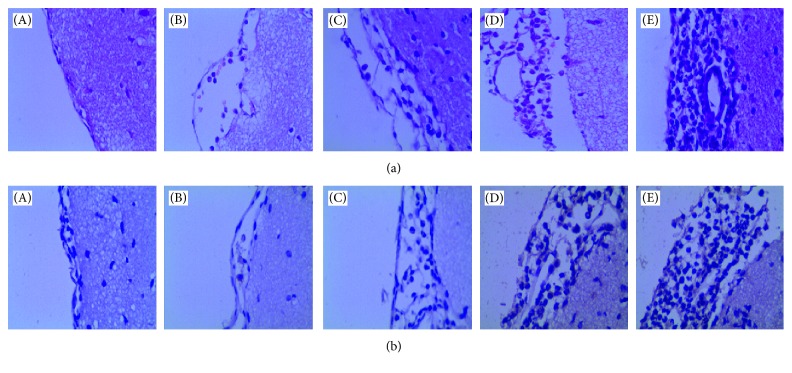
Hematoxylin/eosin-stained and immunohistochemical staining sections (×400). (a) Observe the meningeal structural change of *Streptococcus pneumoniae* meningitis rats by the HE staining method. (b) The meningeal structural change of *Streptococcus pneumoniae* meningitis rats detected by the immunohistochemical method. (A) Control group, (B) 12 h group, (C) 24 h group, (D) 48 h group, and (E) 7 d group.

The structures of brain tissues in the control group were normal. A small amount of neutrophil infiltration could be observed in the 12 h group, along with basically intact vascular wall and mild interstitial oedema. So over time, neutrophil infiltration was increased in the 24 h, 48 h, and 7 d groups, along with interstitial oedema, partial nerve cell degeneration and necrosis, vascular destruction and local bleeding, and necrosis lesions (Figure 3).

### 3.4. NOD2 Immunohistochemical Staining Sections

**Figure 4:**
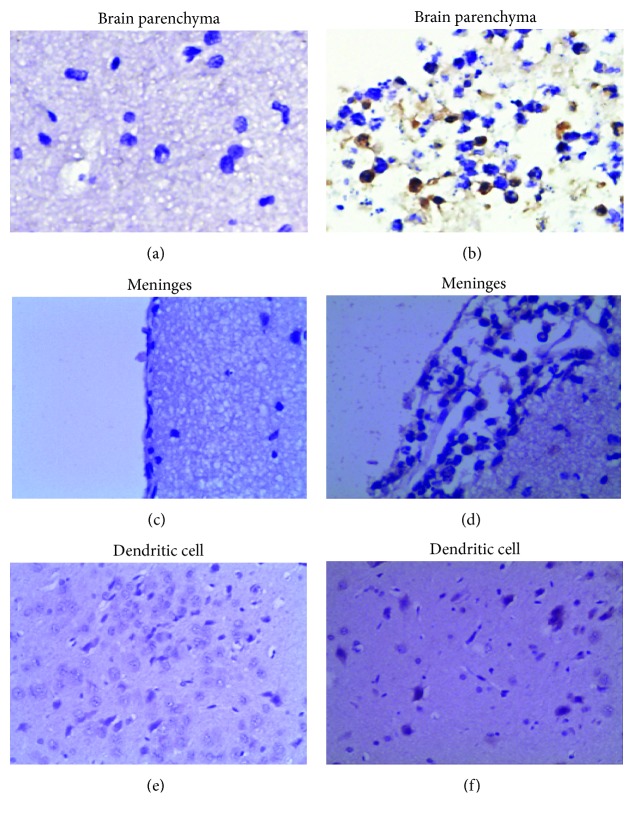
Changes in NOD2 protein expression in brain tissues of rats (IHC, ×400). (a, c, e) The expression of the NOD2 protein is not observed in normal brain tissue. (b, d, f) NOD2 immunopositive cells were widely expressed in the infected Wistar rat meninges, brain parenchyma, and dendritic cells.

There was no positive expression of NOD2 protein in brain tissues of the normal control group. IHC results suggested a positive expression of the NOD2 protein in the infected Wistar rat meninges, brain parenchyma, and dendritic cells: homogeneous diffuse brown color in cell plasm (Figure 4).

### 3.5. Symptoms and Neurological Score in Each Group after Infection in Wistar Rats

**Figure 5:**
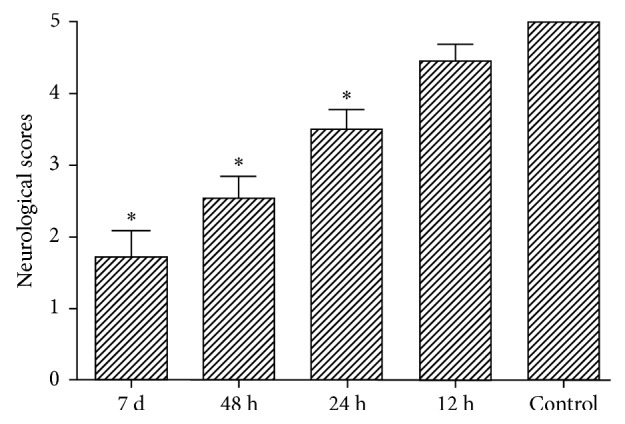
Changes in neurological scores in different groups. The neurological scores were significantly lower in rats with 24 h, 48 h, and 7 d groups compared with the control group. However, no statistically significant interactions between the 12 h group and the control group were found. ^*∗*^*p* < 0.05 versus the control group.

Nervous system symptoms to various degrees occurred in rats after infection with *Streptococcus pneumoniae*, including fatigue, reduced spontaneous activity and food intake, drowsiness, paralysis, ataxia, epileptic seizure, coma, and death. No obvious nervous symptoms were seen in the control group, with the score of 5 points. After infection, the scores in 12 h, 24 h, and 48 h groups, and 7 d group were dropped, which were 4.46 ± 0.23, 3.52 ± 0.24, 2.53 ± 0.32, and 1.72 ± 0.36, respectively; meanwhile, 24 h group, 48 h group, and 7 d group were lower than those of the control group (*p* < 0.05) and this difference was no longer statistically significant in the group of 12 h and control (Figure 5).

### 3.6. Changes in BBB Permeability and BWC after *Streptococcus pneumoniae* Infection

**Figure 6:**
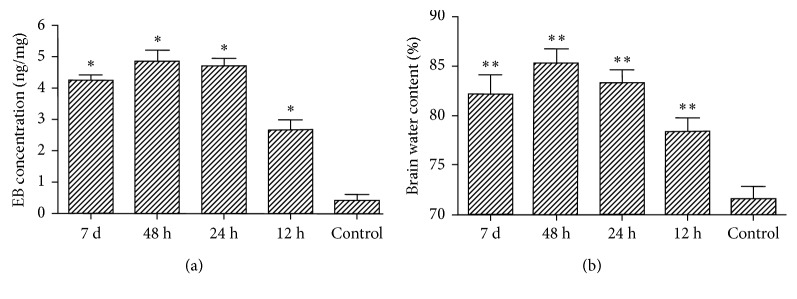
Changes in BBB permeability and BWC in brain tissues. (a) Effect of *Streptococcus pneumoniae* on EB extravasations. The amount of EB extravasation began to increase at 12 h after infection, and the peak value appeared at 24 h and 48 h, and then it had been recovering since 7 d after infection (^*∗*^*p* < 0.05 compared with control rats' brains; *n*=5 in each group). (b) Effect of *Streptococcus pneumoniae* on BWC. The variation curve of brain tissue water content was similar to EB extravasations (^*∗∗*^*p* < 0.05 compared with control rats' brains; *n*=5 in each group).

The BBB was destroyed after *Streptococcus pneumoniae* infection, compared with the control group, BBB permeability and BWC in brain tissues increased to reach its maximal value at 24 h and 48 h, and then it had been recovering since 7 d after infection (*p* < 0.05). These results demonstrated that the impairment of the BBB induced by *Streptococcus pneumoniae* in acute episode from 24 h to 48 h was most obvious. The similar variation curve between BBB opening and brain oedema implied that brain oedema might be the result of BBB opening (Figure 6).

## 4. Discussion

*Streptococcus pneumoniae* is one of the common pathogenic bacteria in central nervous system infection, which will induce *Streptococcus pneumoniae* meningitis that has high mortality at present [23]. Moreover, survival is frequently associated with severe neurological impairment [24]. The exact pathogenesis of *Streptococcus pneumoniae* meningitis remains unclear. It thinks that it is associated with immune factors, blood-brain barrier (BBB) damage, and endothelial dysfunction [25, 26].

Recently, the role of NOD2 protein in bacterial infection has attracted increasing attention [27]. NOD2 belongs to the cytoplasmic protein, which lacks the transmembrane function [28]. However, research discovers that NOD2 can be recruited to the plasma membrane to recognise the muramyldipeptide (MDP) in bacterial peptidoglycan, thus exerting its function [29]. Our findings suggest that after successful modelling through posterior cistern injection, the pathological sections reveal that the subarachnoid space, brain parenchyma, and pia mater conform to the characteristics of bacterial meningitis. Figures 4(b) and 4(d) have revealed the positive NOD2 expression in granular cells, which manifests as brown specks. Interestingly, the positive NOD2 expression in dendritic cells of brain tissues is captured in Figure 4(f). Dendritic cell is the controller of body immunity, which is rare in normal brain tissue due to the presence of BBB. In the case of central nervous system infection, the dendritic cell will gather in the brain parenchyma, which participates in regulating the genesis and development of the disease. Where are the dendritic cells derived in the case of central nervous system infection? Are they differentiated from the central nervous system inherent cells or are infiltrated into the central nervous system? In 2009, Chauhan et al. had discovered positive NOD2 expression in microglial cells [16]. In our study, positive NOD2 protein expression is also found in dendritic cells. Therefore, we speculate that dendritic cells may be transformed from microglial cells.

Furthermore, our study also finds that the NOD2 expression is firstly increased 12 h after infection (early stage of *Streptococcus pneumoniae* meningitis) in the *Streptococcus pneumoniae* meningitis model, which is even higher at 24 h and 48 h. In comparison, the expression of IL-1*β*, TNF-*α*, and IL-6 is not markedly changed 12 h after infection, which is markedly increased 24 h and 48 h after infection. These results are consistent with the findings from Yang et al., further verifying that NOD2 activation can induce NF-*κ*B nuclear transfer, which can affect the expression of downstream target genes as a transcription factor [30]. This will further activate a series of inflammatory cytokines, such as tumor necrosis factor (TNF) and interleukin (IL), which will induce the chemotactic movement of inflammatory cells and further release the related inflammatory media to induce inflammatory changes.

BBB permeability and BWC in the infected rats are detected using the Evans blue method, the results of which discover that the BBB permeability and BWC in the 12 h group start to add up and those in 24 h and 48 h groups continue to grow with the high expression of NOD2 and inflammatory cells, resulting in aggravated brain oedema. We assumed that, in the early period of inflammation, a large amount of white blood cell inflow is the cause of neutrophil activation, which will secrete massive nitric oxide and oxygen, damage the surrounding tissues. It is one of the main reasons to cause cytotoxic oedema and nerve injury ensued [31]. The similar variation curve between BBB opening and brain oedema implied that brain oedema may be the result of BBB opening. Disruption of the BBB might be an important mechanism implicated in the pathogenesis of *Streptococcus pneumoniae* meningitis. Consequently, we speculate that high NOD2 expression may destroy the nerve and vascular matrix, and affect the neurovascular integrity, which further promotes inflammatory cell infiltration of the central nervous system, leading to cerebral injury.

Therefore, high NOD2 expression plays a key role in the pathogenesis of *Streptococcus pneumoniae* meningitis, and suppressing NOD2 expression may be the new strategy for treating *Streptococcus pneumoniae* infection.

## Figures and Tables

**Table 1:** Description of reference genes, primer sequences, and amplicon characteristics.

Gene name	Accession number	Primer sequence (5′–3′) forward/reverse	Amplicon size (bp)	Annealing Tm (°C)
NOD2	NM_001106172.1	GGCAACAGTGTAGGTGATAAGGG	150	60
		TAGTGACTTGTTCTTCTCCAGCATC		
GAPDH	NM_017008.4	TTCCTACCCCCAATGTATCCG	281	60
		CATGAGGTCCACCACCCTGTT		

## Data Availability

The data used to support the findings of this study are available from the corresponding author upon request.

## Abbreviations

APAF-1: Apoptotic protease-activating factor-1
BBB: Blood-brain barrier
BWC: Brain water content
CARD: Caspase-associated recruitment domain
CSF: Cerebrospinal fluid
EB: Evans blue
GM-di: GlcNAc-MurNAc dipeptide
GM-Tri_lys_: GlcNAc-MurNAc tripeptide muropeptide
IHC: Immunohistochemistry
IL: Interleukin
IL-1*β*: Interleukin-1 beta
IL-6: Interleukin-6
MDP: Muramyldipeptide
NOD1: Nucleotide-binding oligomerization domain 1
NOD2: Nucleotide-binding oligomerization domain 2
OD: Optical density
PRR: Pattern-recognition receptors
qRT-PCR: Quantitative real-time polymerase chain reaction
R-protein: Disease-resistance proteins
TNF: Tumor necrosis factor
TNF-*α*: Tumor necrosis factor-alpha.
